# Evaluation of Fiber Contamination from Different Microapplicators in Universal Adhesive Systems: A Pilot In Vitro Study

**DOI:** 10.3390/ma19122562

**Published:** 2026-06-13

**Authors:** Flavius-Alexandru Sinitean, Luminița-Maria Nica, Laura-Elena Cîrligeriu, Anca Jivănescu

**Affiliations:** 1 Department of Restorative Dentistry and Endodontics, TADERP Research Center, Faculty of Dentistry, “Victor Babeș” University of Medicine and Pharmacy, Eftimie Murgu Square 2, 300041 Timișoara, Romania; nica.luminita@umft.ro (L.-M.N.); cirligeriu.laura@umft.ro (L.-E.C.); 2 Department of Conservative Dentistry and Endodontics, Advanced and Digital Endodontic, Restorative and Prosthodontic Treatment (TADERP) Research Center, Faculty of Dental Medicine, “Victor Babeș” University of Medicine and Pharmacy, 300041 Timișoara, Romania; jivanescu.anca@umft.ro; 3 Faculty of Dental Medicine, “Victor Babeș” University of Medicine and Pharmacy, Eftimie Murgu Square 2, 300041 Timișoara, Romania; 4 Department of Prosthodontics, TADERP Research Center, “Victor Babeș” University of Medicine and Pharmacy, B-dul Revoluției 1989, No. 9, 300041 Timișoara, Romania

**Keywords:** universal adhesive, microapplicators, fiber-like contamination, adhesive dentistry, active application, in vitro study

## Abstract

Fiber contamination originating from disposable dental microapplicators has received limited attention despite its potential influence on adhesive procedures. The aim of this pilot in vitro study was to evaluate fiber-like structure release associated with different microapplicator types during the application of universal adhesive systems. Three universal adhesives (Clearfil Universal Bond Quick, Gluma Universal, and G-Premio BOND) and five microapplicator types (X-Slim, Clinique, Prima, Single TIM, and ZerofloX silicone-bristle microapplicators) were evaluated. A total of 75 adhesive applications were performed on standardized sandblasted glass substrates under controlled laboratory conditions. Adhesives were actively applied for 10 s, and fiber-like structures were quantified microscopically using ImageJ software. Statistical analysis included descriptive statistics, two-way ANOVA, and Tukey post hoc testing (α = 0.05). Significant differences were observed among microapplicator types. X-Slim applicators produced the highest fiber counts, whereas Single TIM applicators demonstrated substantially lower values. No detectable fiber-like structures were observed in specimens treated with the ZerofloX silicone-bristle microapplicator. Adhesive system type showed a comparatively smaller influence on fiber counts than microapplicator design. Within the limitations of this pilot in vitro study, microapplicator type appeared to be the primary factor influencing visible fiber contamination during adhesive application. Further studies are required to determine whether the contamination patterns observed influence adhesive performance under clinically relevant conditions.

## 1. Introduction

Advances in adhesive dentistry have enabled the development of minimally invasive restorative procedures and have improved the predictability of enamel and dentin bonding. Universal adhesive systems, also referred to as multimode adhesives, have gained widespread clinical acceptance because they can be used with etch-and-rinse, self-etch, or selective-etch strategies while maintaining simplified clinical protocols [1,2,3,4].

Despite these advances, contamination control remains a critical factor influencing the quality and durability of the adhesive interface. Numerous studies have demonstrated that contamination by saliva, blood, or moisture may adversely affect adhesive procedures and compromise bonding effectiveness [5,6,7]. Consequently, contamination remains one of the major variables affecting the predictability of adhesive procedures.

Most available investigations have focused on biological contaminants; however, considerably less attention has been directed toward iatrogenic contamination originating from the instruments used during adhesive application. Microapplicators are routinely employed during restorative procedures because they facilitate controlled material delivery and active adhesive application. Conventional microapplicators generally consist of synthetic fibers attached to a plastic core and are intended for single use [8].

Microapplicator design may influence flexibility, deformation resistance, and adhesive delivery characteristics. Furthermore, detached fiber-like structures may be observed during the manipulation of conventional fiber-based microapplicators. These fiber-like structures may become incorporated into the uncured adhesive layer, thereby representing a potential source of interfacial contamination. Although this phenomenon has been documented microscopically, its direct influence on bond strength, hybrid layer quality, restoration longevity, or other clinically relevant outcomes remains insufficiently understood. Since the integrity of the adhesive interface is considered a major determinant of long-term restoration performance, any foreign material incorporated within the adhesive layer may potentially influence interfacial behavior [9,10,11].

In response to these concerns, alternative applicator designs have recently been introduced. Alternative silicone-bristle microapplicators have been introduced with the aim of reducing fiber shedding while maintaining adequate handling characteristics; however, comparative evidence remains limited.

Given the scarcity of quantitative data regarding microapplicator-related contamination, the present investigation was designed as a pilot in vitro study. The purpose of the study was to generate preliminary quantitative data, estimate effect sizes, and provide a foundation for future investigations involving larger sample sizes and clinically relevant outcome measures.

Therefore, the aim of the present pilot in vitro study was to evaluate and compare the number of fiber-like structures detected following the application of universal adhesive systems using different microapplicator types under standardized laboratory conditions. The null hypotheses were that: (1) no significant differences would exist among the evaluated microapplicator types regarding fiber counts; (2) adhesive system type would not influence fiber counts; and (3) no interaction would exist between microapplicator type and adhesive system.

## 2. Materials and Methods

### 2.1. Study Design

This pilot in vitro comparative study was designed to evaluate fiber-like structure release associated with different dental microapplicator types during the active application of universal adhesive systems. The study was conceived as a pilot investigation because limited quantitative data are currently available regarding microapplicator-related contamination under standardized adhesive application conditions. The primary objective was to generate preliminary data, estimate effect sizes, and provide a foundation for future investigations involving larger sample sizes and clinically relevant outcome measures.

A factorial experimental design was employed, combining five microapplicator types with three universal adhesive systems. Five independent repetitions were performed for each microapplicator–adhesive combination, resulting in a total of 75 experimental observations. All experimental procedures, including adhesive application, image acquisition, and image analysis, were performed by a single operator to improve protocol standardization and reduce operator-related variability.

### 2.2. Materials

#### 2.2.1. Adhesive Systems

Three commercially available universal adhesive systems were included in this study: Clearfil Universal Bond Quick (Kuraray Noritake Dental Inc., Tokyo, Japan), a universal adhesive compatible with both self-etch and etch-and-rinse application strategies; Gluma Universal (Kulzer GmbH, Hanau, Germany), a multimode universal adhesive; and G-Premio BOND (GC Corporation, Tokyo, Japan), a light-cured universal adhesive compatible with self-etch, selective-etch, and etch-and-rinse strategies.

For each specimen, a standardized drop of adhesive was dispensed directly from the manufacturer’s bottle by holding the bottle in a vertical position and allowing a single drop to fall naturally onto the experimental surface, without the application of external pressure. This approach was used to improve consistency across samples.

The adhesive was intentionally left uncured to allow direct microscopic visualization and quantification of fiber-like structures present within the adhesive layer immediately after application. Although this methodology does not fully reproduce clinical conditions, it enabled direct assessment of contamination generated during adhesive application. All procedures were performed under orange-filtered illumination to prevent premature polymerization.

The evaluated materials were obtained through routine commercial procurement channels and were used according to the manufacturers’ recommendations. Product lot numbers and expiration dates were not systematically recorded at the time of the investigation and are therefore unavailable.

#### 2.2.2. Microapplicators

Five types of disposable dental microapplicators were evaluated in this study: Prima Bonding microapplicators—Size L (Prima Dental Group, Gloucester, UK); Clinique microapplicators Regular (Clinique, Geneva, Switzerland); X-Slim Black Microapplicators (Dr. Mayer, Munich, Germany); Single TIM Adhesive Applicators (VOCO GmbH, Cuxhaven, Germany); and ZerofloX™ Medmix Microapplicators (Medmix, Switzerland).

The first four systems featured conventional fiber-based applicator tips, whereas the ZerofloX™ Microapplicator utilized a silicone-bristle design, allowing comparison between fiber-based and non-fiber applicator technologies.

**Figure 1:**
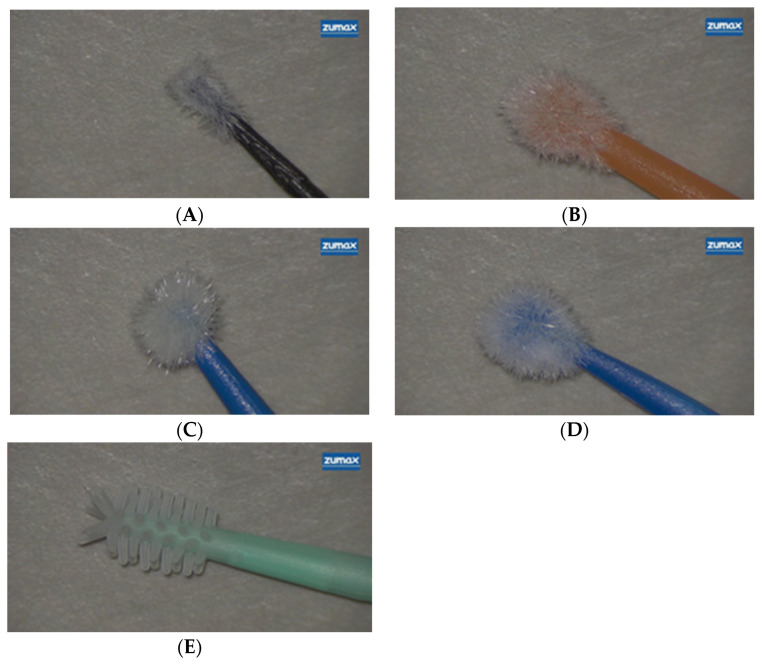
Representative magnified images of the evaluated microapplicator tips obtained using a dental operating microscope prior to adhesive application. (**A**) Prima Bonding Microapplicator (Prima Dental Group, Gloucester, UK); (**B**) Clinique Microapplicators Regular (Clinique, Geneva, Switzerland); (**C**) X-Slim Black Microapplicator (Dr. Mayer, Germany); (**D**) Single TIM Adhesive Applicator (VOCO GmbH, Cuxhaven, Germany); and (**E**) ZerofloX™ Medmix Microapplicator (Medmix, Haag, Switzerland). Images were acquired using a Zumax OMS 2360 dental operating microscope (Zumax Medical Co., Suzhou, China) at an approximate total magnification of ×16.

Prior to testing, representative specimens from each microapplicator type were documented under microscopic magnification to evaluate tip morphology and identify visible surface irregularities before adhesive application (Figure 1A–E). Most applicator tips appeared clean and free of visible impurities, whereas the Clinique Microapplicators Regular exhibited microscopically visible particulate irregularities on the fiber tips.

All evaluated materials were independently purchased through routine commercial channels. No products, materials, financial support, or other forms of sponsorship were provided by the manufacturers.

#### 2.2.3. Experimental Surface

A double-faced glass pad (65 × 135 × 15 mm) was used as the experimental substrate. One surface was smooth, whereas the opposite surface was sandblasted. The sandblasted surface was selected to provide a standardized microretentive substrate that partially resembles the irregular topography of etched enamel while minimizing biological variability associated with dental hard tissues.

No preliminary measurements were performed on enamel or dentin substrates. Consequently, the quantitative contamination values obtained in the present investigation should not be directly extrapolated to clinical substrates.

A circular application area with a diameter of 10 mm was defined using a standardized silicone template to ensure consistency among specimens. The opposite side of the glass plate was protected with adhesive tape to prevent unintended material contact.

### 2.3. Application Protocol

For each experimental condition, one drop of adhesive was dispensed onto the sandblasted surface by holding the adhesive bottle approximately 1 cm above the substrate in a vertical position and allowing a single drop to fall naturally, without applying external pressure.

Active adhesive application was performed using a disposable microapplicator held perpendicular (90°) to the surface. Application was standardized at 10 s for all specimens using a timer. During this period, continuous circular brushing movements were performed within the predefined 10 mm diameter area.

A light manual pressure was applied by a single operator; however, the exact force was not instrumentally measured. During the 10 s application period, approximately 15 circular brushing cycles were performed within the predefined analysis area. Because application pressure was not instrumentally standardized, its potential influence on fiber release cannot be excluded.

Each microapplicator was used only once and discarded after a single application. Five independent applications were performed for each microapplicator–adhesive combination. With five microapplicator types and three adhesive systems, a total of 75 applications were evaluated.

Minor peripheral spreading of the adhesive beyond the template margins was occasionally observed; however, microscopic evaluation remained restricted to the predefined 10 mm analysis area.

All procedures were carried out under orange-filtered microscope illumination to prevent premature adhesive polymerization. Immediately after application, specimens were examined in situ without repositioning or transfer. Only a few seconds elapsed between completion of the application procedure and image acquisition, corresponding to the time required for focusing and adjustment.

All procedures were performed at room temperature (approximately 22 °C). All materials were stored at room temperature in their original packaging before use.

### 2.4. Microscopic Evaluation

Microscopic evaluation was performed using a Zumax OMS 2360 dental operating microscope (Zumax Medical Co., Suzhou, China) equipped with a 250 mm focal length objective lens and 12.5× eyepieces. Image acquisition was performed at objective position 3, corresponding to an approximate total magnification of ×16. All specimens were examined at a standardized working distance determined by the focal length of the objective lens.

Immediately after adhesive application, high-resolution images and continuous video recordings were obtained without repositioning the specimens. Orange-filtered coaxial illumination was maintained throughout the observation period to prevent premature polymerization.

The analysis focused on the presence and number of fiber-like structures located within the predefined 10 mm circular application area. Fiber-like structures were defined as elongated filamentous structures morphologically consistent with detached microapplicator fibers. Dust particles, air bubbles, scratches, adhesive irregularities, and optical artifacts were excluded from counting based on their morphology and appearance under magnification.

**Figure 2:**
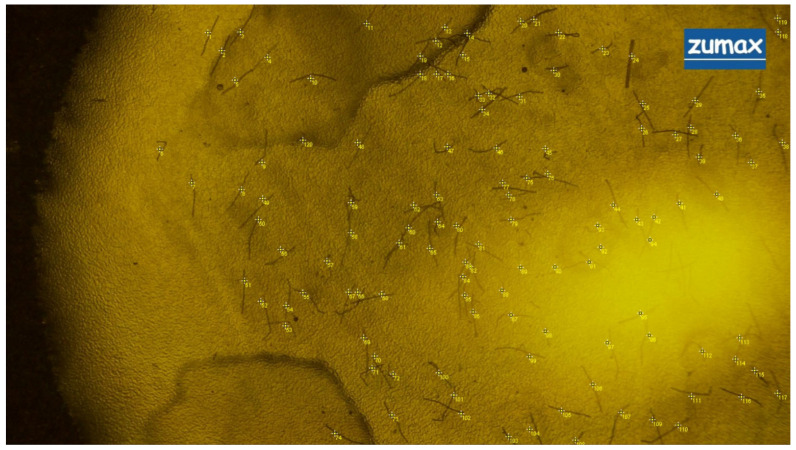
Representative ImageJ-assisted quantification procedure. Individual fiber-like structures identified within the predefined 10 mm analysis area were manually marked and counted using ImageJ software (version 1.54, National Institutes of Health, Bethesda, MD, USA). The numerical labels indicate the structures included in the counting procedure. Non-filamentous features such as dust particles, air bubbles, scratches, and optical artifacts were excluded from analysis based on predefined morphological criteria. The image corresponds to the X-Slim Black Microapplicator (Dr. Mayer, Germany) used with Clearfil Universal Bond Quick (Kuraray Noritake Dental Inc., Tokyo, Japan).

Fiber quantification was performed using ImageJ software, version 1.54 (National Institutes of Health, Bethesda, MD, USA) (Figure 2). Every visible fiber-like structure identified within the predefined analysis area was manually counted and recorded. Fiber counts were subsequently aggregated for descriptive and statistical analysis.

Image analysis was performed by a single evaluator who was also responsible for specimen preparation and image acquisition. Consequently, the evaluator was not blinded to the experimental groups. Repeated counting and formal intra-observer reliability assessment were not performed. These factors represent limitations of the present pilot investigation and should be considered when interpreting the results.

All procedures were conducted under standardized experimental conditions to improve reproducibility.

### 2.5. Statistical Analysis

Statistical analysis was performed to evaluate the effects of microapplicator type, adhesive system, and their interaction on fiber counts. The outcome variable was defined as the number of fiber-like structures detected within the standardized 10 mm diameter application area following a single adhesive application.

Descriptive statistics were calculated for each microapplicator–adhesive combination and expressed as mean, standard deviation, minimum value, and maximum value. Additional descriptive analyses were performed after pooling data according to microapplicator type and adhesive system.

Normality of residual distribution was assessed using the Shapiro–Wilk test, whereas homogeneity of variances among microapplicator–adhesive combinations was evaluated using Levene’s test. The results of these assumption tests are reported in the Section 3. Because the present investigation was designed as an exploratory pilot study and employed a balanced factorial design with an equal number of observations in each experimental group, a two-way analysis of variance (ANOVA) was used to evaluate the effects of microapplicator type, adhesive system, and their interaction on fiber counts.

When statistically significant differences were identified, pairwise comparisons were performed using Tukey’s honestly significant difference (HSD) post hoc test. Effect sizes were reported as partial eta squared (η^2^). All tests were two-tailed, and statistical significance was established at *p* < 0.05.

Although Levene’s test indicated heterogeneity of variances, the study employed a balanced factorial design with equal sample sizes across all groups. Therefore, two-way ANOVA was retained as the primary inferential approach. The results should nevertheless be interpreted within the limitations of this exploratory pilot study.

Given the count-based nature of the outcome variable and the presence of a zero-inflated group (ZerofloX™), the statistical findings should be interpreted as exploratory and confirmed in future studies using alternative analytical approaches.

To further evaluate the robustness of the findings and account for the count-based nature of the outcome variable and the presence of a zero-inflated group (ZerofloX™), an additional non-parametric sensitivity analysis was performed using the Kruskal–Wallis test. This analysis was used to verify whether the observed differences among microapplicator groups remained significant independently of the assumptions required for parametric testing.

## 3. Results

### 3.1. Overview of Experimental Design

A total of 75 measurements were analyzed, corresponding to five independent applications for each microapplicator–adhesive combination. The outcome variable was defined as the number of fiber-like structures identified within the predefined 10 mm diameter analysis area following a single adhesive application.

The ZerofloX™ Medmix silicone-bristle microapplicator consistently showed no detectable fiber-like structures across all evaluated adhesive systems.

### 3.2. Qualitative Microscopic Observations

Microscopic evaluation revealed the presence of fiber-like structures within the uncured adhesive layer for all evaluated fiber-based microapplicators. In contrast, no detectable fiber-like structures were identified in specimens treated with the ZerofloX™ Medmix silicone-bristle microapplicator under the experimental conditions used.

**Figure 3:**
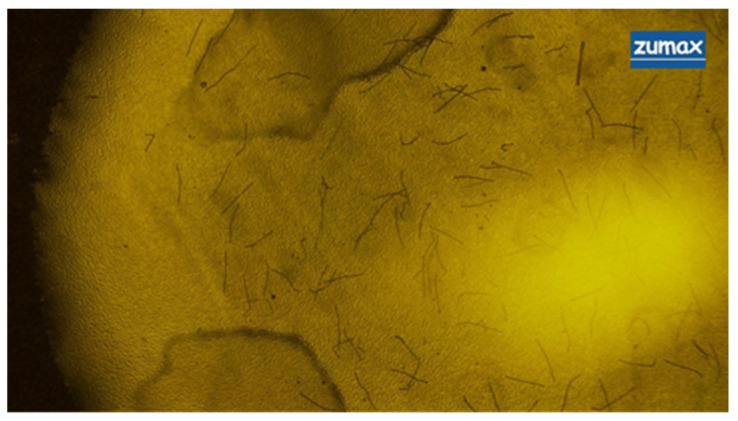
Representative microscopic image showing extensive visible fiber-like contamination produced by X-Slim Black Microapplicators, characterized by a high density and clustering of fiber-like structures within the analysis area. Bright scattered particles visible in the image were not classified as fiber-like structures and were excluded from analysis.

**Figure 4:**
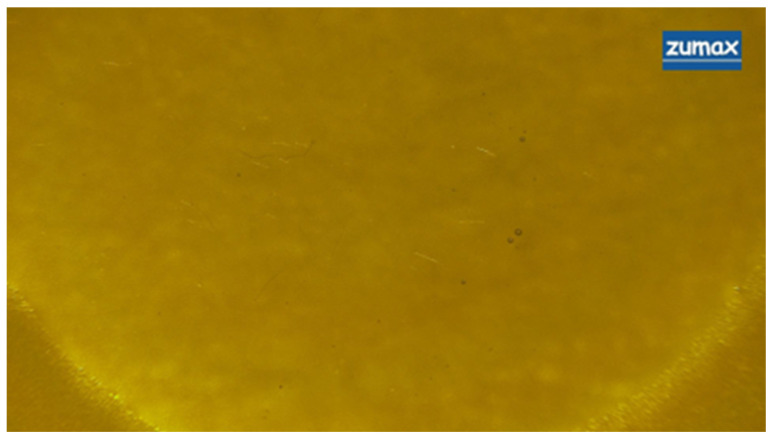
Representative microscopic image obtained following application with the Single TIM Adhesive Applicator and G-Premio BOND (GC Corporation, Tokyo, Japan), showing sparse and predominantly isolated fiber-like structures. Any bright scattered particles visible in the image were not classified as fiber-like structures and were excluded from analysis.

Distinct contamination patterns were observed among the evaluated microapplicators. X-Slim Black Microapplicators frequently exhibited dense accumulations of fiber-like structures distributed throughout the analysis area, often forming overlapping configurations and clusters (Figure 3). In contrast, Single TIM Adhesive Applicators generally exhibited fewer fiber-like structures that appeared predominantly isolated and more sparsely distributed (Figure 4).

**Figure 5:**
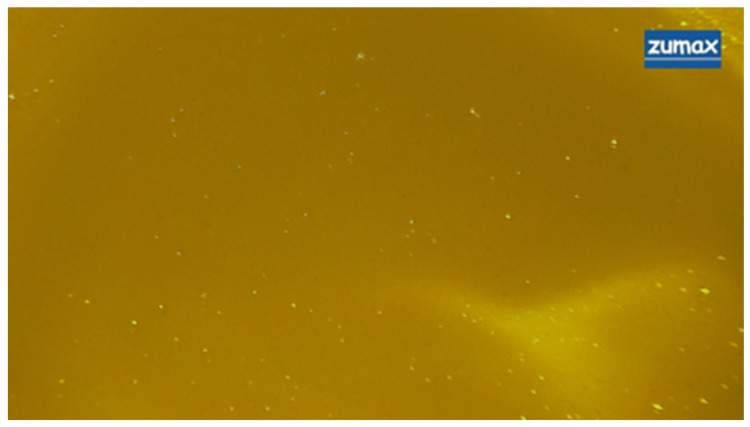
Representative microscopic image obtained following application with the ZerofloX™ Medmix silicone-bristle microapplicator and Clearfil Universal Bond Quick, showing no detectable fiber-like structures under the experimental conditions used. Bright scattered particles visible in the image were not classified as fiber-like structures and were excluded from analysis.

Representative images obtained following application with the ZerofloX™ Medmix silicone-bristle microapplicator did not reveal detectable fiber-like structures within the evaluated area (Figure 5).

No major qualitative differences in fiber morphology were observed among the three adhesive systems tested. Similar contamination patterns were observed across repeated applications.

The Single TIM Adhesive Applicator exhibited fewer fiber-like structures than the other evaluated fiber-based microapplicators. Fiber-like structures appeared predominantly as isolated structures, with minimal clustering and a sparse distribution pattern (Figure 4).

No detectable fiber-like structures were identified in specimens treated with the ZerofloX™ Medmix silicone-bristle microapplicator under the experimental conditions used (Figure 5).

### 3.3. Descriptive Statistics

Table 1 presents descriptive statistics for all microapplicator–adhesive combinations. Values represent the number of fiber-like structures identified within the predefined 10 mm diameter analysis area following a single adhesive application.

The highest mean fiber counts were observed for the X-Slim Black Microapplicators combined with Clearfil Universal Bond Quick (116.6 ± 9.8 fiber-like structures per application), whereas the lowest mean values among fiber-based applicators were observed for the Single TIM Adhesive Applicators combined with G-Premio BOND (15.0 ± 3.1 fiber-like structures per application). All ZerofloX™ Medmix specimens exhibited zero detectable fiber-like structures.

### 3.4. Effect of Microapplicator Type

Table 2 summarizes fiber counts according to microapplicator type after pooling data from all adhesive systems.

The highest mean fiber count was observed for the X-Slim Black Microapplicators (101.4 ± 14.3), followed by Clinique Microapplicators Regular (64.8 ± 7.1) and Prima Bonding Microapplicators (47.1 ± 8.1). Single TIM Adhesive Applicators exhibited substantially lower mean fiber counts (15.9 ± 3.2), whereas no detectable fiber-like structures were observed for the ZerofloX™ Medmix silicone-bristle microapplicator.

### 3.5. Effect of Adhesive System

Table 3 summarizes fiber counts according to adhesive system after pooling data from all microapplicator groups.

Mean fiber counts were 49.9 ± 41.2 for Clearfil Universal Bond Quick, 45.8 ± 36.9 for Gluma Universal, and 41.8 ± 32.8 for G-Premio BOND. The differences observed among adhesive systems were smaller than those observed among microapplicator types.

### 3.6. Two-Way ANOVA

Prior to inferential analysis, the assumptions required for parametric testing were evaluated. The Shapiro–Wilk test applied to the ANOVA residuals did not indicate a significant deviation from normality (W = 0.974, *p* = 0.117), whereas Levene’s test demonstrated heterogeneity of variances among the microapplicator–adhesive combinations (F = 2.388, *p* = 0.010). These findings were considered during interpretation of the results and represent an inherent limitation of the present pilot study.

A two-way analysis of variance (ANOVA) was performed to evaluate the effects of microapplicator type and adhesive system on fiber counts. A statistically significant effect of microapplicator type was observed (F(4,60) = 525.09, *p* < 0.001, partial η^2^ = 0.972), indicating that microapplicator design exerted a substantial influence on fiber counts.

A statistically significant effect of adhesive system was also identified (F(2,60) = 9.54, *p* < 0.001, partial η^2^ = 0.241). In addition, a statistically significant interaction between microapplicator type and adhesive system was detected (F(8,60) = 4.11, *p* < 0.001, partial η^2^ = 0.354).

These findings indicate that microapplicator type represented the dominant factor influencing fiber counts, whereas adhesive system exerted a comparatively smaller effect under the experimental conditions used.

### 3.7. Non-Parametric Sensitivity Analysis

Given the count-based nature of the outcome variable and the presence of a zero-inflated group, an additional non-parametric sensitivity analysis was performed using the Kruskal–Wallis test. The analysis confirmed statistically significant differences among the evaluated microapplicator types (H(4) = 70.67, *p* < 0.001). These findings were consistent with the results obtained from the two-way ANOVA, supporting the robustness of the observed differences among microapplicator groups despite the limitations associated with the distribution of the data.

### 3.8. Post Hoc Analysis (Tukey HSD)

Pairwise comparisons using Tukey’s honestly significant difference (HSD) test demonstrated significantly higher fiber counts for the X-Slim Black Microapplicators compared with all other evaluated microapplicator groups (*p* < 0.001).

Clinique Microapplicators Regular exhibited significantly higher fiber counts than Prima Bonding Microapplicators and Single TIM Adhesive Applicators (*p* < 0.05). Prima Bonding Microapplicators also demonstrated significantly higher fiber counts than Single TIM Adhesive Applicators (*p* < 0.05).

Single TIM Adhesive Applicators exhibited significantly lower fiber counts than all conventional fiber-based microapplicators (*p* < 0.05), whereas the ZerofloX™ Medmix silicone-bristle microapplicator demonstrated significantly lower fiber counts than all other evaluated groups (*p* < 0.001).

### 3.9. Representative Microscopic Images

**Figure 6:**
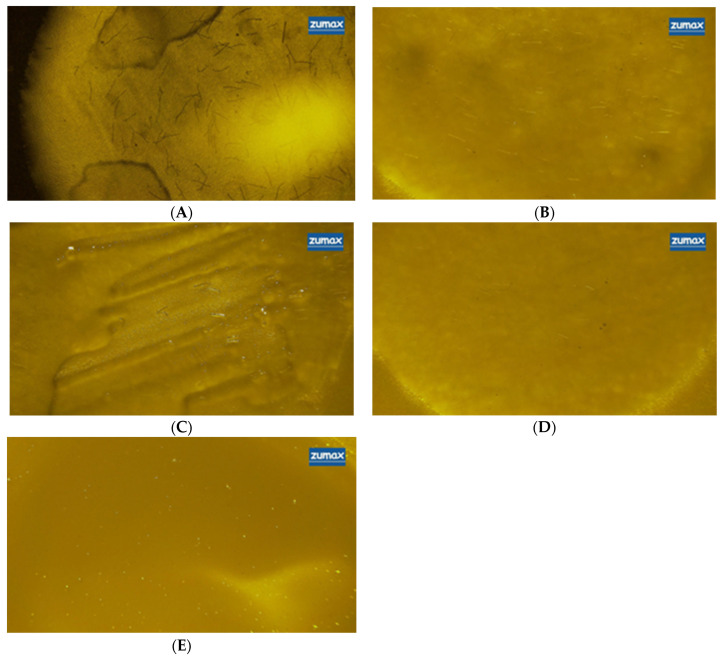
Representative comparison of the five evaluated microapplicator types following application of Clearfil Universal Bond Quick under standardized experimental conditions. (**A**) X-Slim Black Microapplicator; (**B**) Clinique Microapplicator Regular; (**C**) Prima Bonding Microapplicator; (**D**) Single TIM Adhesive Applicator; and (**E**) ZerofloX™ Medmix silicone-bristle microapplicator. Visible fiber-like contamination varied substantially among the fiber-based microapplicators, whereas no detectable fiber-like structures were observed for the ZerofloX™ applicator under the present experimental conditions.

Representative microscopic images illustrating the contamination patterns associated with each microapplicator type are presented in Figure 6. Distinct differences were observed regarding fiber density, distribution, and clustering among the evaluated applicators. X-Slim Black Microapplicators exhibited dense accumulations of fiber-like structures, whereas Single TIM Adhesive Applicators demonstrated sparse and predominantly isolated structures. No detectable fiber-like structures were observed in specimens treated with the ZerofloX™ Medmix silicone-bristle microapplicator.

## 4. Discussion

### 4.1. Influence of Microapplicator Design

The most important finding of the present study was the substantial variation in fiber counts among the evaluated microapplicator types. All conventional fiber-based microapplicators produced detectable fiber-like structures within the adhesive layer, although the magnitude of contamination differed considerably among products. In contrast, the tested ZerofloX™ silicone-bristle microapplicator showed no detectable fiber-like structures under the experimental conditions used.

X-Slim Black Microapplicators exhibited the highest mean fiber counts, whereas Single TIM Adhesive Applicators demonstrated substantially lower values. These findings suggest that structural differences among fiber-based applicators may influence the tendency for visible fiber release during active adhesive application. The findings of the present study suggest that applicator architecture may contribute to contamination patterns through differences in flexibility, deformation resistance, and adhesive delivery characteristics.

The lower fiber counts observed for the Single TIM applicator may be related to differences in fiber arrangement, fiber density, or manufacturing characteristics; however, these parameters were not directly evaluated in the present study. Similarly, the absence of detectable fiber-like structures in the ZerofloX™ group suggests that silicone-bristle designs may reduce visible fiber contamination. Nevertheless, because only one silicone-bristle microapplicator system was evaluated, caution should be exercised when extrapolating these findings to other non-fiber applicator systems.

Overall, the present results indicate that microapplicator design may influence contamination patterns during adhesive procedures. The robustness of these findings was further supported by the non-parametric sensitivity analysis, which also demonstrated significant differences among microapplicator types. This suggests that the observed contamination patterns were not solely dependent on the assumptions associated with parametric statistical testing.

### 4.2. Influence of Adhesive Systems

Although statistically significant differences among adhesive systems were identified, the magnitude of these differences was substantially smaller than the effect associated with microapplicator type. Clearfil Universal Bond Quick exhibited slightly higher mean fiber counts than Gluma Universal and G-Premio BOND.

Several material-related factors, including viscosity, wettability, solvent composition, and surface interaction characteristics, have been reported to influence the behavior of adhesive materials [2,10,12,13]. However, these properties were not directly measured in the present investigation. Consequently, no causal relationship between adhesive composition and fiber retention can be established based on the available data.

The present findings therefore suggest that adhesive system type may exert a secondary influence on fiber counts, whereas microapplicator design appeared to be the dominant factor under the experimental conditions used. This observation is consistent with previous reports emphasizing that operator- and instrument-related variables may significantly influence adhesive procedures despite advances in adhesive chemistry [7].

### 4.3. Interaction Between Microapplicator Type and Adhesive System

A statistically significant interaction between microapplicator type and adhesive system was identified. This finding indicates that the effect of a given adhesive system was not entirely independent of the applicator used.

Differences among adhesive systems were more apparent in groups exhibiting higher fiber counts, whereas comparatively small variations were observed in groups associated with lower contamination levels. Although the mechanisms underlying this interaction remain unclear, the findings suggest that contamination patterns may result from a combination of applicator-related and material-related factors.

Because the present study was not designed to investigate the physicochemical mechanisms responsible for this interaction, further research is required to clarify the factors involved. Similar interactions between material characteristics and application variables have previously been reported in studies evaluating adhesive procedures and restorative materials [13].

### 4.4. Microscopic Characteristics of Fiber Contamination

Microscopic evaluation revealed distinct differences in fiber density, distribution, and clustering among the evaluated microapplicator types. X-Slim Black Microapplicators frequently exhibited dense accumulations of fiber-like structures and overlapping configurations, whereas Single TIM Adhesive Applicators generally demonstrated fewer and more isolated structures. No detectable fiber-like structures were observed in specimens treated with the ZerofloX™ silicone-bristle microapplicator.

The present microscopic observations demonstrated the presence of fiber-like structures within adhesive layers and highlighted the potential for instrument-related contamination during adhesive procedures. The present observations provide a qualitative description of contamination patterns associated with different applicator designs. However, the potential influence of these patterns on adhesive interface quality, bond strength, restoration longevity, or other clinically relevant outcomes was not evaluated and therefore cannot be inferred from the present data.

### 4.5. Potential Clinical Relevance

Although the present study did not directly evaluate adhesive performance, the findings highlight the possibility that disposable microapplicators may represent a previously underappreciated source of contamination during adhesive procedures.

Structural irregularities and defects within adhesive interfaces have been associated with degradation phenomena in previous investigations [9,14,15,16]. However, the present study did not assess whether the fiber-like structures identified influenced adhesive penetration, hybrid layer formation, bond strength, nanoleakage, marginal adaptation, polymerization quality, or restoration longevity.

Consequently, the clinical significance of the observed contamination remains uncertain. The present findings should therefore be interpreted as evidence of visible fiber-like structure release rather than evidence of impaired adhesive performance. Future studies incorporating clinically relevant outcome measures are necessary to determine whether the contamination patterns observed have measurable biological or mechanical consequences.

### 4.6. Limitations of the Study

Several limitations should be acknowledged. First, this was a pilot in vitro study performed on a standardized sandblasted glass substrate rather than enamel or dentin. Although this approach minimized biological variability and improved standardization, the findings cannot be directly extrapolated to clinical substrates.

Second, the adhesive layer was intentionally left uncured to facilitate direct microscopic visualization and quantification of fiber-like structures. Therefore, the possibility that polymerization may influence the visibility or retention of fiber-like structures cannot be excluded. While this methodology enabled contamination assessment, it does not fully reproduce clinical conditions in which adhesive systems are light-cured.

Third, image analysis was performed by a single evaluator who was also responsible for specimen preparation and image acquisition. Although the application duration and approximate number of brushing cycles were standardized, application pressure was not instrumentally controlled and may have influenced fiber release. Consequently, the evaluator was not blinded to the experimental groups. Repeated counting and formal intra-observer reliability assessment were not performed, representing additional methodological limitations.

Fourth, several material-related properties that may potentially influence contamination patterns, including viscosity, wettability, and solvent-related characteristics, were not directly measured.

Fifth, the exact volume of a single adhesive drop was not measured for each adhesive system; therefore, potential differences in dispensed adhesive volume cannot be completely excluded.

Finally, deviations from normality and homogeneity of variance were observed, and the sample size was limited by the exploratory pilot design. Consequently, the findings should be interpreted as preliminary and confirmed in future investigations involving larger sample sizes and alternative statistical approaches. Although a non-parametric sensitivity analysis yielded results consistent with the primary ANOVA model, future studies involving larger sample sizes and count-based statistical models (e.g., Poisson or negative binomial regression) would provide a more comprehensive evaluation of the observed contamination patterns.

### 4.7. Future Perspectives

Future studies should investigate whether the fiber-like structures observed in the present study influence adhesive interface characteristics, bond strength, nanoleakage, marginal integrity, or long-term restoration performance. Investigations employing enamel and dentin substrates, polymerized adhesive layers, and higher-resolution imaging techniques may further clarify the clinical relevance of microapplicator-related contamination.

In addition, comparative evaluation of alternative applicator designs may contribute to the development of adhesive delivery systems that minimize visible contamination while maintaining favorable handling characteristics.

## 5. Conclusions

Within the limitations of this pilot in vitro study, microapplicator type was identified as the primary factor influencing the number of fiber-like structures detected during the application of universal adhesive systems. All evaluated fiber-based microapplicators exhibited detectable fiber-like structures, although substantial differences were observed among products.

Among the evaluated applicators, X-Slim Black Microapplicators exhibited the highest mean fiber counts, whereas Single TIM Adhesive Applicators exhibited comparatively lower values. The tested ZerofloX™ silicone-bristle microapplicator showed no detectable fiber-like structures under the experimental conditions used.

Although statistically significant differences among adhesive systems were identified, their influence was considerably smaller than the effect associated with microapplicator type. The mechanisms responsible for these differences were not directly investigated and therefore cannot be determined from the present data.

The findings suggest that microapplicator selection may influence visible contamination patterns during adhesive application. However, the present study did not evaluate whether the observed contamination patterns influence bond strength, hybrid layer formation, nanoleakage, marginal adaptation, polymerization quality, or long-term restoration performance. Consequently, the clinical significance of the observed contamination remains uncertain.

Further investigations employing clinically relevant outcome measures are required to determine whether the contamination patterns observed in the present study influence adhesive performance or restoration longevity.

## Figures and Tables

**Table 1:** Fiber counts per application for each microapplicator–adhesive combination (mean ± SD; n = 5).

Microapplicator	Adhesive	Mean ± SD	Min	Max
X-Slim Black Microapplicators (Dr. Mayer, Germany)	Clearfil Universal Bond Quick (Kuraray Noritake Dental Inc., Tokyo, Japan)	116.6 ± 9.8	107	129
X-Slim Black Microapplicators (Dr. Mayer, Germany)	Gluma Universal (Kulzer GmbH, Hanau, Germany)	99.8 ± 11.3	84	113
X-Slim Black Microapplicators (Dr. Mayer, Germany)	G-Premio BOND (GC Corporation, Tokyo, Japan)	87.8 ± 6.4	81	97
Prima Bonding microapplicators—Size L (2.0 mm, blue) (Prima Dental Group, Gloucester, UK)	Clearfil Universal Bond Quick (Kuraray Noritake Dental Inc., Tokyo, Japan)	52.2 ± 9.6	40	66
Prima Bonding microapplicators—Size L (2.0 mm, blue) (Prima Dental Group, Gloucester, UK)	Gluma Universal (Kulzer GmbH, Hanau, Germany)	45.4 ± 6.7	38	56
Prima Bonding microapplicators—Size L (2.0 mm, blue) (Prima Dental Group, Gloucester, UK)	G-Premio BOND (GC Corporation, Tokyo, Japan)	43.8 ± 7.5	36	56
Clinique microapplicators Regular (Clinique, Geneva, Switzerland)	Clearfil Universal Bond Quick (Kuraray Noritake Dental Inc., Tokyo, Japan)	64.2 ± 6.3	55	73
Clinique microapplicators Regular (Clinique, Geneva, Switzerland)	Gluma Universal (Kulzer GmbH, Hanau, Germany)	68.0 ± 8.0	56	75
Clinique microapplicators Regular (Clinique, Geneva, Switzerland);	G-Premio BOND (GC Corporation, Tokyo, Japan)	62.2 ± 6.8	51	70
Single TIM Adhesive Applicators (VOCO GmbH, Cuxhaven, Germany)	Clearfil Universal Bond Quick (Kuraray Noritake Dental Inc., Tokyo, Japan)	16.6 ± 3.9	12	23
Single TIM Adhesive Applicators (VOCO GmbH, Cuxhaven, Germany)	Gluma Universal (Kulzer GmbH, Hanau, Germany)	16.0 ± 2.6	13	20
Single TIM Adhesive Applicators (VOCO GmbH, Cuxhaven, Germany)	G-Premio BOND (GC Corporation, Tokyo, Japan)	15.0 ± 3.1	10	18
ZerofloX™ Medmix Microapplicators (Medmix, Switzerland)	Clearfil Universal Bond Quick (Kuraray Noritake Dental Inc., Tokyo, Japan)	0 ± 0	0	0
ZerofloX™ Medmix Microapplicators (Medmix, Switzerland)	Gluma Universal (Kulzer GmbH, Hanau, Germany)	0 ± 0	0	0
ZerofloX™ Medmix Microapplicators (Medmix, Switzerland)	G-Premio BOND (GC Corporation, Tokyo, Japan)	0 ± 0	0	0

**Table 2:** Mean fiber counts according to microapplicator type after pooling data from all adhesive systems. Values represent the number of fiber-like structures identified per application.

Microapplicator	Mean ± SD
X-Slim Black Microapplicators (Dr. Mayer, Germany)	101.4 ± 14.3
Prima Bonding microapplicators—Size L (2.0 mm, blue) (Prima Dental Group, Gloucester, UK)	47.1 ± 8.1
Clinique microapplicators Regular (Clinique, Geneva, Switzerland)	64.8 ± 7.1
Single TIM Adhesive Applicators (VOCO GmbH, Cuxhaven, Germany)	15.9 ± 3.2
ZerofloX™ Medmix Microapplicators (Medmix, Switzerland)	0 ± 0

**Table 3:** Mean fiber counts according to adhesive system after pooling data from all microapplicator groups. Values represent the number of fiber-like structures identified per application.

Adhesive	Mean ± SD
Clearfil Universal Bond Quick (Kuraray Noritake Dental Inc., Tokyo, Japan)	49.9 ± 41.2
Gluma Universal (Kulzer GmbH, Hanau, Germany)	45.8 ± 36.9
G-Premio BOND (GC Corporation, Tokyo, Japan)	41.8 ± 32.8

## Data Availability

The original contributions presented in this study are included in the article. Further inquiries can be directed to the corresponding author.

## Abbreviations

The following abbreviations are used in this manuscript:

ANOVA	Analysis of Variance
CC BY	Creative Commons Attribution
SD	Standard Deviation
SEM	Scanning Electron Microscopy
